# Supplementary material for: Alternative Splicing Regulation of Low-Frequency Genetic Variants in Exon 2 of TREM2 in Alzheimer’s Disease by Splicing-Based Aggregation
Source: Int J Mol Sci. 2021 Sep 13;22(18):9865. doi: 10.3390/ijms22189865 (PMC8471326; doi:10.3390/ijms22189865)
Supplement: Supplementary file 1 [file ijms-22-09865-s001.zip › ijms-1301857-supplementary.pdf]

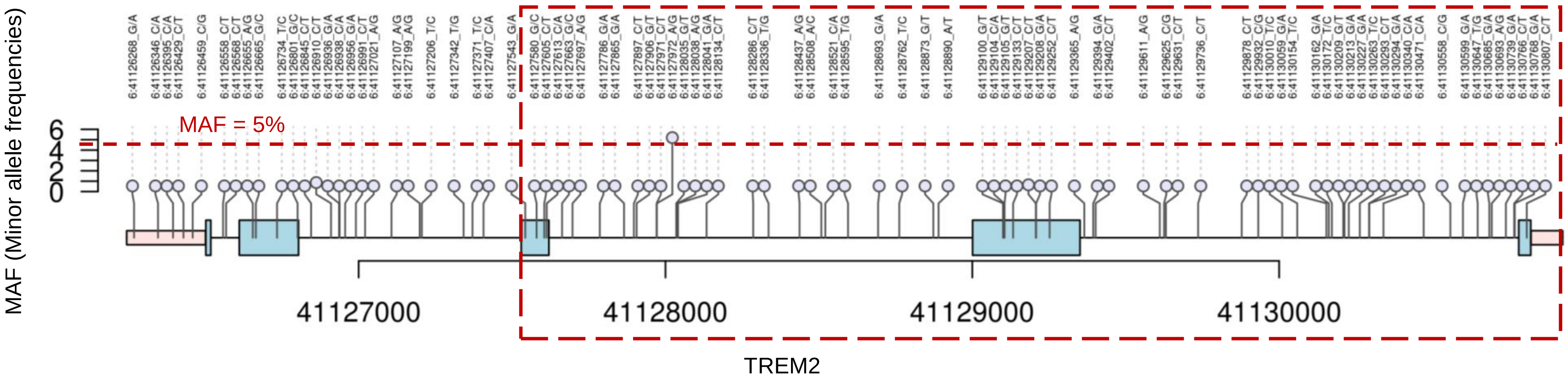

Figure S1. Minor allele frequencies (MAFs) of *TREM2* variants identified in our data. Here, we focus on 63 variants within exon 2 and its neighboring exons (i.e., the red box). The *trackViewer* R package was used for visualization.

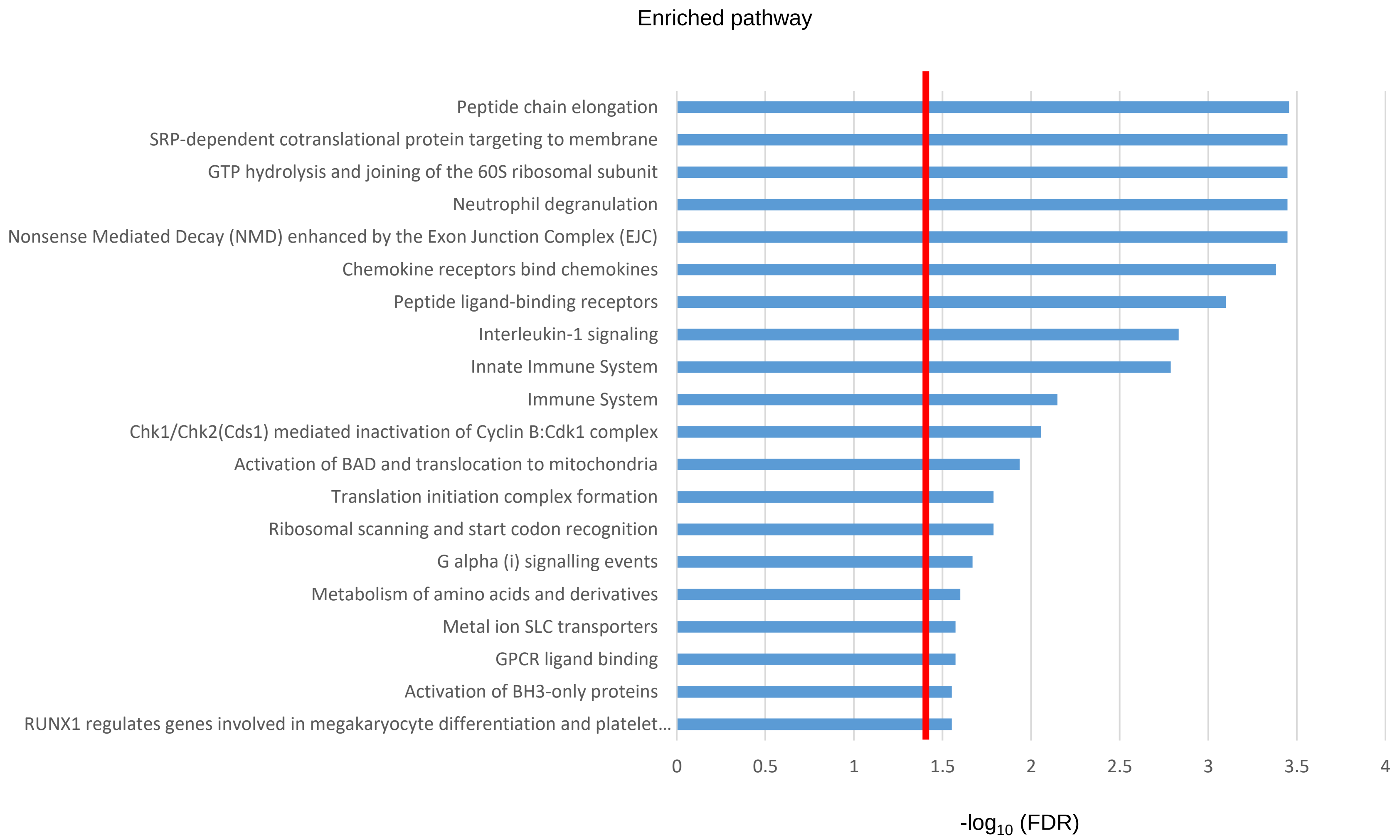

Figure S2. Immune-related functional REACTOME pathways enriched among genes differentially expressed between individuals with low and high skipping of the 2nd exon of *TREM2*. The red vertical line indicates the significant level (i.e., FDR = 0.05).

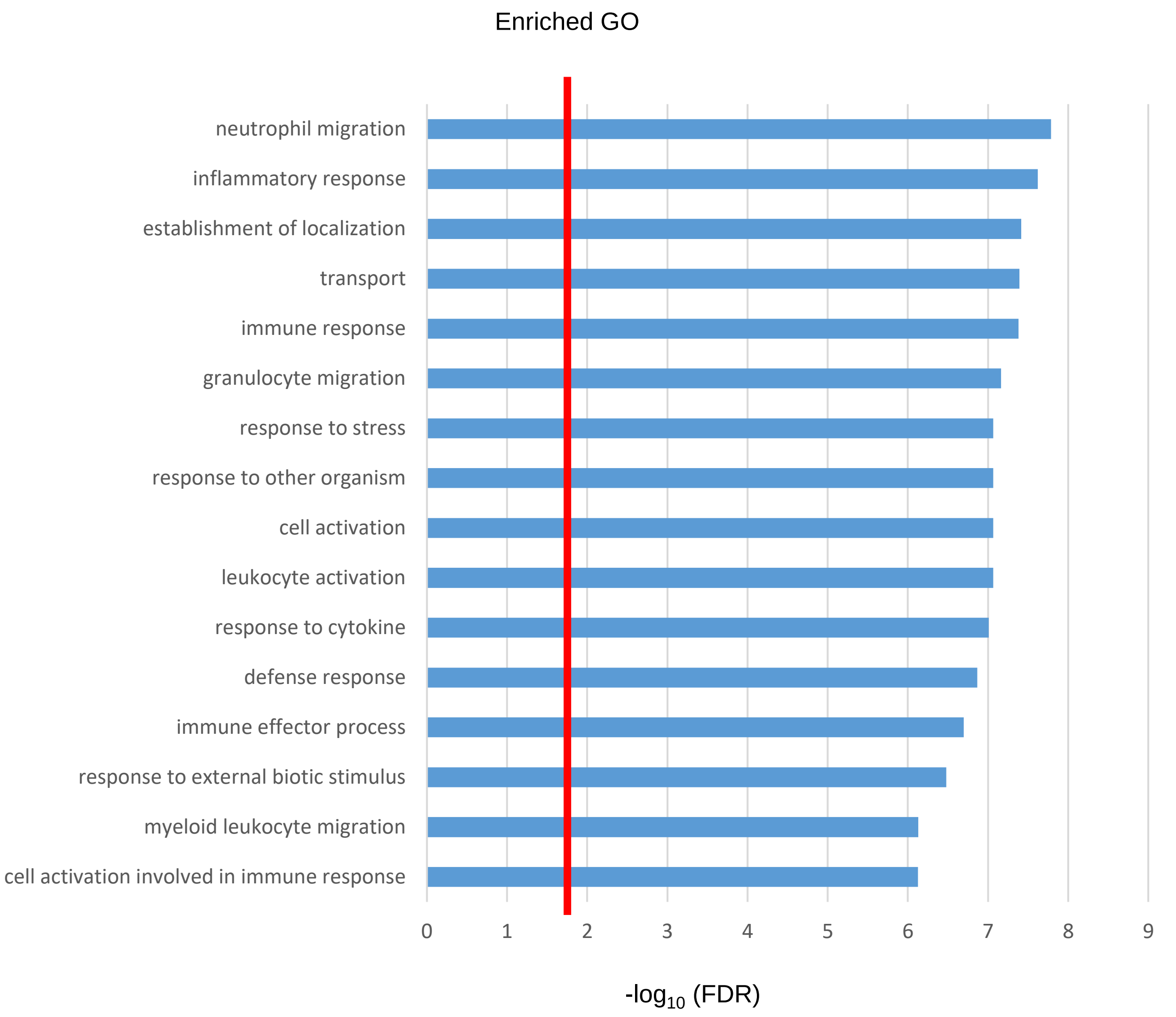

Figure S3. Immune-related GO terms enriched among genes differentially expressed between individuals with low and high skipping of the 2nd exon of *TREM2*. The red vertical line indicates the significant level (i.e., FDR = 0.05).

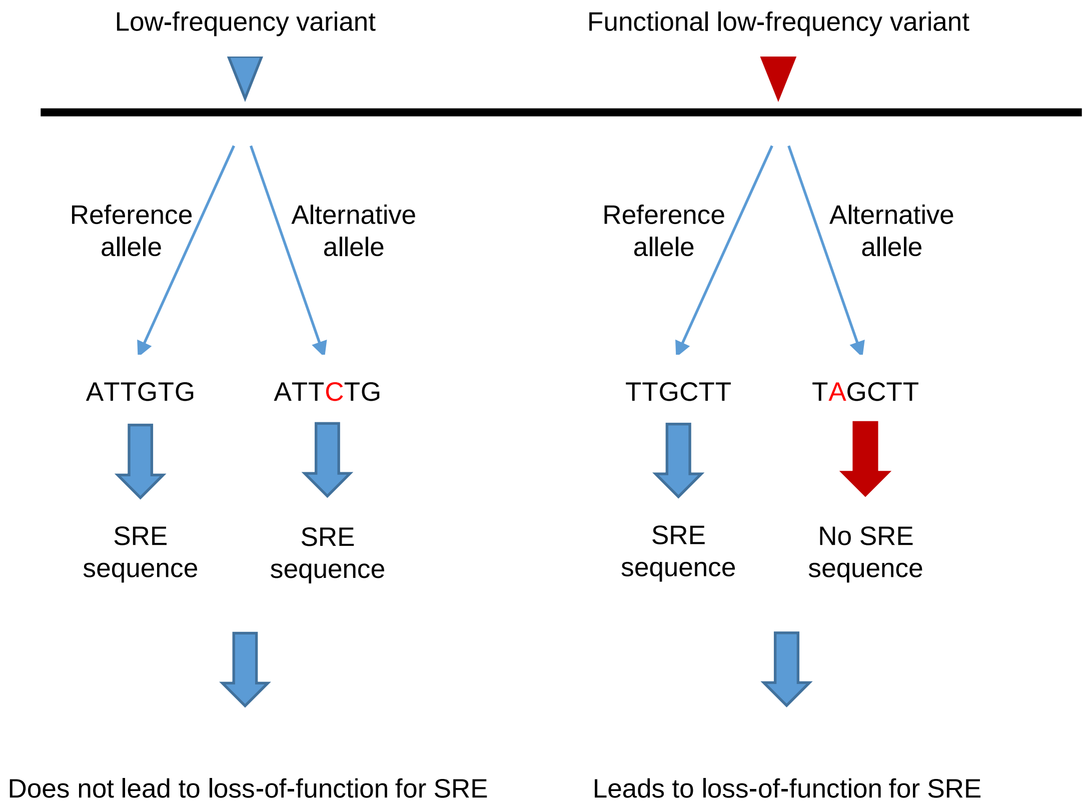

Figure S4. Definition of a functional variant based on SRE context. If the reference genotype matches a SRE sequence, but the alternative allele (i.e., red letter) does not, this variant could lead to loss-of-function of the SRE and is defined as a functional variant.

Table S1. The positions of 10 functional variants within SREs.

rsid	Position	Ref. allele	Alt. allele	Ref. sequence	Alt. sequence	SREs in ref. sequences	SREs in alt. sequences
rs182653531	chr6:41127786	G	A	AGTGG**G**CCGTT	AGTGG**A**CCGTT	AGTGGG, GTGGGC	x
rs115121185	chr6:41127897	C	T	CCTTC**C**GCACA	CCTTC**T**GCACA	CCTTCC	x
rs184276085	chr6:41128762	T	C	GCAGG**T**GATGC	GCAGG**C**GATGC	GCAGGT,CAGGTG,AGGTGA	x
rs936961326	chr6:41128873	G	T	AGTAG**G**GAGAT	AGTAG**T**GAGAT	GTAGGG,TAGGGA,AGGGAG,GGGAGA	x
rs143332484	chr6:41129207	C	T	CCACA**C**GCTGG	CCACA**T**GCTGG	CGCTGG	x
rs201258314	chr6:41129208	G	A	CACAC**G**CTGGC	CACAC**A**CTGGC	CGCTGG	ACACAC,CACACA
rs565502230	chr6:41129932	C	G	AGCCC**C**CTGCT	AGCCC**G**CTGCT	CCCCTG, CCCTGC, CCTGCT	x
rs59377666	chr6:41130162	G	A	GCACC**G**TCTCT	GCACC**A**TCTCT	GTCTCT	x
rs74390253	chr6:41130471	C	A	AGCTT**C**CCCCA	AGCTT**A**CCCCA	CTTCCC,TTCCC,TCCCCC	x
rs369487317	chr6:41130739	G	A	TCAAA**G**TGAGG	TCAAAATGAGG	GTGAGG	x

Red and blue colors indicate an exon skipping enhancer (i.e., ESE and ISE) and siliencer (ESS) in SRE, respectively.

Bold refers a reference or alternative allele of variants.

Table S2. Individuals carrying functional variants.

Disease status	Sex	PSI	Variants
AD	Female	1	chr6:41130162
AD	Female	0.692	chr6:41130162
AD	Female	0.778	chr6:41129207
AD	Female	0.871	chr6:41130162,chr6:41130471
AD	Female	1	chr6:41130162,chr6:41130471
AD	Male	0.778	chr6:41129207
AD	Male	1	chr6:41129207
AD	Female	0.854	chr6:41128873
AD	Female	0.926	chr6:41128873
AD	Female	0.818	chr6:41129208,chr6:41130739
AD	Female	0.8	chr6:41129208,chr6:41130739
AD	Female	0.909	chr6:41129932
AD	Female	0.9	chr6:41129932
AD	Female	0.76	chr6:41128762
AD	Male	0.852	chr6:41130162
AD	Male	0.722	chr6:41130162
AD	Male	0.867	chr6:41130162
AD	Male	0.5	chr6:41129207
AD	Male	1	chr6:41129207
AD	Female	0.75	chr6:41130162,chr6:41130471
AD	Female	0.714	chr6:41130162,chr6:41130471
AD	Male	1	chr6:41130162
AD	Male	0.75	chr6:41129207
AD	Female	0.862	chr6:41129207
AD	Male	0.875	chr6:41129932
AD	Female	0.941	chr6:41129207
AD	Female	1	chr6:41129207
AD	Male	1	chr6:41129207
AD	Female	0.862	chr6:41129207
AD	Male	0.75	chr6:41127786
AD	Female	0.714	chr6:41129207
AD	Female	0.857	chr6:41129207
AD	Female	0.667	chr6:41129207
AD	Female	0.913	chr6:41129207
AD	Female	0.802	chr6:41130162,chr6:41130471
AD	Female	1	chr6:41129207
CN	Male	1	chr6:41127897
CN	Male	0.538	chr6:41130162
CN	Male	0.632	chr6:41130162
CN	Female	1	chr6:41128762
CN	Female	0.895	chr6:41129207
CN	Male	0.833	chr6:41130162
CN	Female	0.939	chr6:41127897
CN	Female	0.875	chr6:41127897

CN	Male	0.98	chr6:41129932
CN	Male	0.829	chr6:41129207
CN	Male	0.667	chr6:41129207
CN	Male	0.8	chr6:41129932
CN	Female	0.949	chr6:41129207
CN	Male	1	chr6:41130739
CN	Female	1	chr6:41130162,chr6:41130471
CN	Female	0.976	chr6:41129207

Table S3. Differentially expressed genes between samples with low- and high-PSI values of exon 2.

GeneID	Esemble transcriptID	FDR	Fold change
IL1B	ENST00000263341	3.26E-12	3.198465919
GPR84	ENST00000267015	5.21E-11	2.45140965
CCL2	ENST00000225831	5.78E-10	2.929687277
FCER1G	ENST00000490414	1.39E-08	2.480163699
ICAM1	ENST00000264832	2.93E-08	2.096651723
BATF	ENST00000286639	4.46E-08	2.398003926
SIGLEC9	ENST00000440804	5.69E-08	2.073651496
SERPINA1	ENST00000393087	5.69E-08	2.036576363
HAMP	ENST00000222304	6.03E-08	2.431166133
CHI3L1	ENST00000472064	1.10E-07	2.037096325
HAMP	ENST00000598398	3.22E-07	2.754895582
SERPINA3	ENST00000482740	8.20E-07	2.31792971
SLC11A1	ENST00000354352	3.09E-06	2.256521841
BCL2A1	ENST00000267953	3.34E-06	2.091704341
CHI3L1	ENST00000478742	5.71E-06	2.08667426
SERPINA3	ENST00000556968	6.71E-06	2.292378595
CHI3L1	ENST00000473185	9.27E-06	2.50604024
SERPINA3	ENST00000556388	1.62E-05	2.174382632
GAPDH	ENST00000492719	3.40E-05	2.052334671
SLC1A5	ENST00000412532	3.42E-05	2.594352603
RUNX1	ENST00000325074	5.46E-05	2.741464209
CP	ENST00000460674	7.28E-05	2.29626851
YWHAH	ENST00000471374	9.94E-05	2.94333831
STAB1	ENST00000462741	0.000106204	2.059575093
YBX3	ENST00000228251	0.00011798	2.094983634
MT1A	ENST00000290705	0.000169901	2.106493946
FOSL1	ENST00000312562	0.000237511	2.478285448
COX7A2	ENST00000472311	0.00024826	2.619067574
C3	ENST00000599668	0.000249871	2.111490292
HSPA6	ENST00000309758	0.000290526	4.670248096
CXCL2	ENST00000508487	0.000581454	2.105298362
GABARAP	ENST00000570856	0.001399893	2.421332063
CLDN11	ENST00000489485	0.001434229	2.405419245
CDK2	ENST00000555408	0.001576344	2.009516361
ARHGEF40	ENST00000553709	0.002697549	-2.032641881
BSCL2	ENST00000421906	0.002699923	2.569483435
SFN	ENST00000339276	0.002724247	2.074913807
CXCL10	ENST00000306602	0.003355138	2.146658958
RPS24	ENST00000464716	0.004342484	2.022765212
HSPA1A	ENST00000608703	0.004356686	2.111602676
CXCL3	ENST00000296026	0.006842996	2.120094775
RPS27	ENST00000392558	0.007375178	2.465661187
IL1R1	ENST00000409589	0.00775483	2.111107838
CXCL2	ENST00000510048	0.008973947	2.075831437

ATP5B	ENST00000551182	0.009391684	2.252858307
PREB	ENST00000456259	0.009451667	2.110922171
TMEM70	ENST00000517439	0.009489252	-2.123820017
CXCL1	ENST00000509101	0.009751183	2.908122121
RPL4	ENST00000566039	0.011236964	2.49606125
NDUFS8	ENST00000528492	0.012092791	2.323440668
FBXL16	ENST00000562563	0.012576076	-3.237091434
RPL36	ENST00000579446	0.013770543	2.370317325
CXCL2	ENST00000296031	0.015065078	2.141763723
SLC1A2	ENST00000606205	0.017024108	-2.239092098
ZNF326	ENST00000394583	0.01882383	2.499926274
MPPE1	ENST00000586364	0.01978445	-2.000986835
KCNC4	ENST00000489935	0.021384463	-2.036712687
MAP2K1	ENST00000425818	0.021485216	-2.179477721
HGS	ENST00000573320	0.021522337	3.354943383
APP	ENST00000474136	0.024360777	-2.233781332
JPH3	ENST00000537256	0.025716855	-2.174434502
TNFSF14	ENST00000245912	0.026668465	2.322433678
RPL37	ENST00000507642	0.028276685	2.12337477
MYH11	ENST00000576790	0.029311117	-2.406307281
CXCL1	ENST00000395761	0.029340761	2.130680929
IGHA1	ENST00000390547	0.033557967	2.004726148
NDRG2	ENST00000557676	0.035148479	2.042849576
GOLM1	ENST00000388711	0.037501247	2.173182221
CCL3	ENST00000225245	0.039130404	2.680096667
TERF2	ENST00000566051	0.041434574	-2.412073475
RPS20	ENST00000519606	0.042653661	2.064298616
VPS51	ENST00000533827	0.042800636	2.62793456
SIN3B	ENST00000595900	0.049904527	-2.343667496

Table S4. Demographic information of analyzed samples.

Region	MSBB		ROSMAP
	FP[a]	IFG[b]	DLPFC[c]
# of AD samples (Male/Female)	175 (53/122)	157 (43/114)	376 (126/250)
# of CN samples (Male/Female)	72 (36/36)	63 (33/30)	213 (82/131)
Male/ Female	89/158	76/144	208/381
Braak stage	3.75 (1.89) [0-6]	3.68 (1.83) [0-6]	4.49 (1.25) [0-6]
APOE	E2E2: 2<br>E2E3: 17<br>E2E4: 1<br>E3E3:84<br>E3E4: 44<br>E4E4: 3<br>Unknown: 96<br>Freq.[d] of E4: 0.168	E2E2: 2<br>E2E3: 15<br>E2E4: 1<br>E3E3:73<br>E3E4: 37<br>E4E4: 3<br>Unknown: 89<br>Freq. of E4: 0.167	E2E2: 5<br>E2E3: 77<br>E2E4: 13<br>E3E3:360<br>E3E4: 129<br>E4E4: 5<br>Freq. of E4: 0.129

a. FP. Frontal pole
b. IFG. Inferior frontal gyrus
c. DLPFC. Dorsolateral prefrontal cortex
d. Freq. Frequency
